# A survey of international medical volunteers’ experiences of working with Voluntary Service Overseas in Ethiopia

**DOI:** 10.1177/0049475518794723

**Published:** 2018-08-20

**Authors:** Mary McCauley, Yohannes Amado, Nynke van den Broek

**Affiliations:** 1 Senior Clinical Research Associate, Centre for Maternal and Newborn Health, Liverpool School of Tropical Medicine, Liverpool, UK; 2 Health Programme Manager, Voluntary Service Overseas, Addis Ababa, Ethiopia; 3 Head, Centre for Maternal and Newborn Health, Liverpool School of Tropical Medicine, Liverpool, UK

**Keywords:** Healthcare providers, low-income countries, volunteering

## Abstract

Many skilled medical professionals from high-income countries volunteer to work in poor-resource settings. There is, however, little research to assess the views and experiences of such healthcare providers. Our study sought to explore this among Voluntary Service Overseas volunteers working in Ethiopia for one year. An online survey was distributed to all returned international medical volunteers one year after their return to their base country. Most felt that they had impacted the local community in which they worked and reported a positive experience, recommending this to friends or family, but there was a small subgroup whose experience was otherwise. We believe there is a need for more comprehensive, systematic and robust monitoring in order to evaluate the outcomes of medical volunteers’ placements.

## Introduction

Sustainable Development Goal #3 (SDG3) is to ensure healthy lives and promote wellbeing for all at all ages.^1^ A lack of trained healthcare providers is one of the barriers to achieving SDG3 in many low- and middle-income countries (LMICs).^1^ Sub-Saharan Africa has 11% of the world’s population and 24% of the global burden of disease, yet only 3% of the world’s healthcare providers, equating to < 2.5 healthcare providers per 1000 population.^2^ Many skilled medical professionals (nurses, midwives, doctors) from high-income countries recognise that they have a responsibility to support healthcare delivery in poorer countries and thus engage in voluntary work.^3^

There are many types of healthcare volunteer placements: short term (e.g. two weeks) with the aim to provide emergency medical aid; or long term (e.g. one year or more) with the aim to build capacity, train staff and strengthen health systems. Voluntary Services Overseas (VSO) is an international development organisation which works to create sustainable change in communities with international, national and youth volunteers. Within the health sector, VSO has worked with 141 health partners across 18 LMICs to train 23,500 healthcare providers to improve their clinical knowledge and practical skills. It is estimated that > 867,000 adults and children have received health services that VSO has supported.^4^

There is evidence that volunteering benefits not only the community into which a volunteer works but also affects the individual volunteer.^5,6^ However, little research has been conducted among healthcare providers. We, therefore, conducted a survey to assess the views and experiences of all medical volunteers working for one year as full-time healthcare providers partnering with VSO in Ethiopia.

## Methods

We emailed a questionnaire in September 2014 to all 17 healthcare providers who had worked as medical volunteers with VSO in Ethiopia for one year. We developed a ten-point questionnaire from a larger survey previously used by VSO.^7^ For ease of completion, all questions were closed-ended and questions were not linked to previous answers. The questionnaire could not be submitted if any of the questions were not answered. Data were analysed using Excel 2013. Full institutional ethical approval for the study was not necessary as we examined the personal views of medical volunteers via an anonymised short online questionnaire. Implied consent was given through the completion of each survey.

## Results

The response rate was 94% with 16/17 medical volunteers responding. International medical volunteers were recruited from the UK (n = 9), Ireland (n = 4) and Canada (n = 3). The cadre of medical volunteers were nurses (n = 2), midwives (n = 3) and doctors (n = 11) including paediatricians, obstetricians, general practitioners, physicians and anaesthetists. Most volunteers were contracted for 12 months but the majority (81.2%) left after the minimum period of time (eight months). Three volunteers (18.8%) extended their contracts.

The majority of medical volunteers reported that they had the required skills for the placement; felt that their work impacted the local community; and could monitor and report on the progress of their placement. Several volunteers agreed that the local partner was a good choice but less than half thought their placement was a good investment and use of resources. The majority of medical volunteers felt that the success of their placement depended primarily on the effectiveness of the working relationship with their local partner. Many volunteers reported there was a lack of understanding or difference in expectations regarding the aims and objectives of the placement between the medical volunteer and the local partner. Only half of the medical volunteers were satisfied with the relationship they had with the programme manager and the programme support staff in the VSO Ethiopia office. Half of all the medical volunteers felt the work they had performed was valued by VSO Ethiopia and only a few volunteers felt that their concerns had been addressed appropriately in a timely manner by the country office staff.

When asked ‘What do you think were the most pertinent obstacles to overcome in your placement?’ and answering ‘yes’ or ‘no’, many respondents replied ‘yes’ to discrepancy between local partner and volunteer expectations (68.8%), low staff morale in the local setting (68.8%), inadequate clinical infrastructure (68.8%), lack of essential equipment and resources (43.8%), gender issues such as a male-dominated work environment and undermining of female input (43.8%), corruption (43.8%) and dealing with difficult clinical scenarios such as potentially preventable deaths of patients (50%). When asked ‘Would you recommend volunteering with this organisation to friends, family and/or other?’, 75% responded ‘yes’ (25%, 50% ‘probably’), 6.3% ‘not sure’ and 18.7% ‘no, probably not’.

## Discussion

### Statement of principal findings

Our survey provides an insight into challenges faced by medical volunteers working through partnerships in clinical health systems in low-resource settings. The majority of medical volunteers were doctors from the UK, who reported a positive experience and would recommend volunteering to their friends or family. However, there was a small subgroup who would not.

### Strengths and limitations

To the best of our knowledge, ours is the first survey to date exploring returned medical volunteers’ views and experiences partnering with VSO in Ethiopia. This survey provides views of volunteers at a specific time only. There was no option for respondents to give free text feedback. With a small sample size (n = 16) it may not be possible to make any further generalisations. Furthermore, these data may only be applicable to medical volunteers working in Ethiopia and may not be related to other types of volunteers working in other countries. However, there was a high (94%) response rate in this survey. A reason for this may be that the author is a returned medical volunteer and was known to all the other volunteers. The fact that volunteers were asked to participate in a questionnaire from a peer may have increased the response rate and we acknowledge that there may be potential bias here. The sample size was too small to assess the effect of sex, personal experience and type of healthcare provider on the reflections of medical volunteer experiences.

### Results of study in relation to other literature

There are largely anecdotal reports in the literature that describe effects of volunteering. Reported positive effects of volunteering in general include an increase in the following: cultural sensitivity; global awareness; adaptability; interpersonal skills; handling responsibility; stress management; confidence; self-assurance; problem solving; team working; management; leadership; and strategic thinking.^6,7^ The results from this study are similar to a study that VSO conducted to survey all returned VSO volunteers.^7^ As part of this evaluation, 2735 volunteers completed a closed-ended online survey, of whom 35% were education volunteers and 14% were health volunteers. Of all the volunteers who completed the survey (n = 2735), many reported benefits such as an increased adaptability (86%), new knowledge and learning (84%), increased confidence (84%), changes in attitude (83%), new skills (82%) and increased resilience (82%).^7^ Other studies focusing on medical volunteers have highlighted that medical volunteers report personal satisfaction and that they had made a difference in the lives of patients as well as the local healthcare providers.^9–11^ These positive effects may translate as benefit in volunteers’ base or home communities and countries.

**Figure 1.**
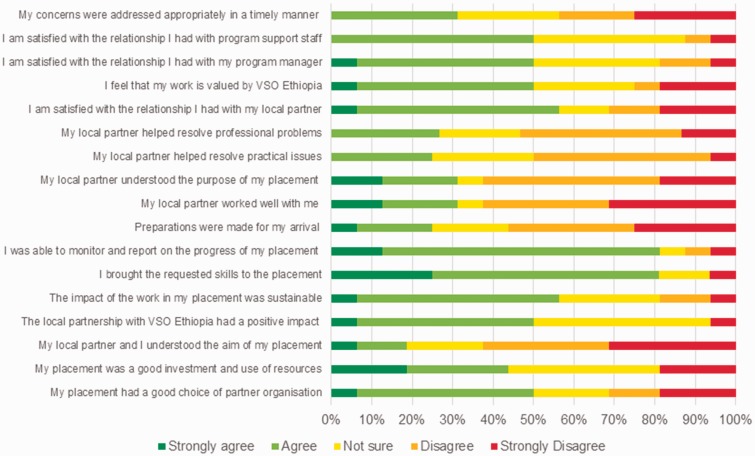
To what extent do you agree with the following statements?

**Figure 2.**
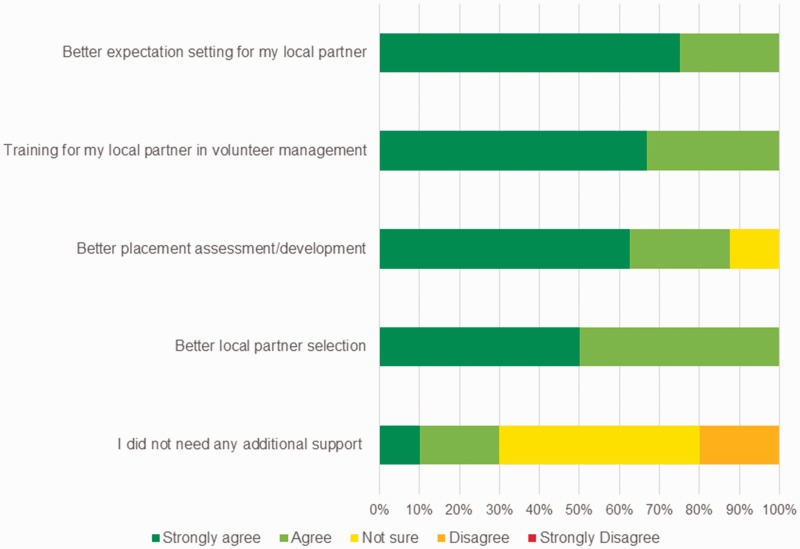
How could the country office staff have better supported you to be more effective in your placement?

However, there are reports of negative effects involved with volunteering. In this study nearly half of all respondents reported challenges such as dealing with difficult clinical scenarios, lack of essential equipment and resources, gender issues and perceived corruption in their placements. Many volunteers in this survey also reported that low staff morale and an inadequate clinical infrastructure were barriers to a successful healthcare placement. These results are similar to the larger VSO survey, in which all types of volunteers reported feeling frustrated (76%), lonely (61%), overwhelmed (55%), stressed (52%), demotivated (35%) and a loss in confidence (33%). In this larger VSO survey, other common challenges reported included difficulties with the host and/or partner organisation (66%), poor infrastructure (57%), corruption (51%) and challenges with the in-country VSO office (48%).^7^

### Implications for healthcare providers and policy-makers

Medical volunteering is not straightforward and each individual volunteer has a unique and personal experience. Working in any country with a different culture and language can be a demanding and stressful experience for many volunteers; this survey has demonstrated that this experience can be even more challenging for medical volunteers working in resource-poor clinical settings. VSO does provide comprehensive support in each base country, including online and residential weekend courses for outgoing and returning volunteers. However, there need to be more support systems (e.g. example mentorship programs) for medical volunteers in the host country, both from local partners and in country staff. This survey also demonstrates that more attention needs to be paid to the choice of local partners, emphasising good management and leadership skills and a clear understanding of the objectives of the volunteer’s placements.

### Unanswered questions and future research

There is a need for qualitative research to explore further the views and experiences of medical volunteers from high-income countries working with international development organisations in clinical settings in a broader range of LMIC settings. Further research should investigate both the positive and negative effects of volunteering on the individual person and should also explore the views and experiences of the local partners. There is currently a lack of literature describing rigorous monitoring and evaluation systems to assess the effectiveness of medical volunteering placements; there is also a lack of consensus on best practice on how to conduct and support both short- and long-term effective medical volunteer placements in low-resource settings. It would be beneficial to understand better how the sharing of expertise between different health systems can be facilitated and how this sharing is supportive and sustainable over time between countries.
